# High Sensitivity Troponins Discriminate Different Morphologies of Coronary Artery Plaques Being Assessed by Coronary Computed Tomography Angiography

**DOI:** 10.1155/2017/9306409

**Published:** 2017-07-18

**Authors:** Jonas Rusnak, Michael Behnes, Nadine Reckord, Ursula Hoffmann, Michèle Natale, Julia Hoffmann, Kathrin Weidner, Siegfried Lang, Agnibh Mukherji, Mathieu Kruska, Thomas Henzler, Stefan O. Schoenberg, Martin Borggrefe, Thomas Bertsch, Ibrahim Akin

**Affiliations:** ^1^First Department of Medicine, University Medical Center Mannheim, University of Heidelberg, Mannheim, Germany; ^2^DZHK (German Center for Cardiovascular Research) Partner Site, Mannheim, Germany; ^3^Institute of Clinical Radiology and Nuclear Medicine, Faculty of Medicine Mannheim, University Medical Center Mannheim (UMM) and Heidelberg University, Mannheim, Germany; ^4^Institute of Clinical Chemistry, Laboratory Medicine and Transfusion Medicine, General Hospital Nuremberg and Paracelsus Medical University, Nuremberg, Germany

## Abstract

**Background:**

This study evaluates the association between high sensitivity troponin I (hsTnI) and T (hsTnT) and the morphology of coronary artery plaques detected by coronary computed tomography angiography (CCTA) in patients with suspected coronary artery disease (CAD).

**Methods:**

Patients undergoing CCTA were prospectively enrolled. CCTA was indicated by a low to intermediate pretest probability for CAD during routine clinical care. Within 24 hours of CCTA examination, peripheral blood samples were taken to measure hsTnI, hsTnT, and N-terminal probrain natriuretic peptide (NT-proBNP).

**Results:**

A total of 99 patients were enrolled with 43% without CAD, 9% with noncalcified plaques, 28% with calcified plaques, and 19% with mixed type plaque lesions. Both hsTnI and hsTnT levels were able to discriminate significantly between the groups, especially in the presence of mixed coronary plaques (AUC range: 0.741–0.752; *p* = 0.0001). In multivariate logistic regression models, hsTnT, but not hsTnI, was still significantly associated with mixed coronary plaque morphology (odds ratio = 8.968; 95% CI 1.999–40.241; *p* = 0.004).

**Conclusions:**

Both hsTnI and hsTnT are able to discriminate between different coronary artery plaques morphologies, whereas hsTnT was significantly associated with mixed coronary plaques in patients with suspected CAD. This trial is registered with NCT03074253.

## 1. Introduction

From a clinical perspective, cardiac troponins represent the main diagnostic parameter to diagnose patients suffering from an acute coronary syndrome (ACS). Contemporary troponin assays aim to detect the release of troponins from cardiomyocytes undergoing ischemia or necrosis. In the past decades, high sensitivity (hs) troponin assays were developed, which might be able to detect even the presence of subclinical coronary artery disease (CAD). Several studies demonstrated the potential value of hs troponins for screening of CAD or risk prediction for adverse cardiovascular events in patients with stable CAD [1–5].

An acute ST-segment elevation myocardial infarction (STEMI) is a consequence of acute coronary arterial thrombosis at the site of a vulnerable ruptured coronary plaque. Therefore, the composition of coronary arterial plaque might represent to be even more important compared to its stenosing character. Histologically, a coronary plaque is prone to rupture when possessing a thin fibrous cap, a large necrotic core, and a high content of macrophages [6, 7]. Detecting those lesions has emerged to an important diagnostic aim during routine clinical care. Modern intravascular imaging techniques, such as intravascular ultrasound (IVUS) and optical coherence tomography (OCT), are able to invasively visualize plaque composition and provide the closest match with histopathologically proven plaque morphology [8, 9].

Coronary computed tomography angiography (CCTA) has become the main noninvasive imaging technique being able to detect the presence of CAD or to predict adverse coronary events [10–12]. However, due to the observation that CCTA overestimates the severity of CAD [13], it is mostly used to exclude CAD in patients presenting with typical, atypical, or nonanginal pain plus a low to intermediate pretest probability (PTP) for CAD according to European guidelines (PTP of 15%–50%; class of recommendation IIa; level of evidence A) [14].

Moreover, CCTA might also be able to characterize the morphology of coronary plaques [15, 16]. Previous studies demonstrated a correlation between the amount of calcium and the atherosclerotic plaque volume, presuming that calcium scoring is a reliable marker for extensive atherosclerosis [17, 18]. According to the “Providing Regional Observations to Study Predictors of Events in the Coronary Tree” (PROSPECT) study, Xu et al. confirmed that calcium correlates with plaque burden being assessed by IVUS [19]. Hs troponin assays have been previously evaluated as a valuable screening parameter for the presence of CAD and prognostic assessment for upcoming adverse cardiovascular events [20–22]. As well as hs troponins, N-terminal probrain natriuretic peptide (NT-proBNP) is able to predict upcoming adverse cardiovascular events in patients with stable CAD [23]. NT-proBNP was evaluated in patients with stable angina pectoris and showed a close correlation to CAD severity as assessed by coronary angiography [24].

However, whether hs troponin levels might reflect the presence of different plaque morphologies has not been well investigated. Therefore, this study aims to evaluate associations between hs troponin I (hsTnI) and hs troponin T (hsTnT) and CCTA-based coronary plaque morphology in patients at low to intermediate CAD risk. Additionally, the study evaluates whether combining both hs troponins with NT-proBNP reveals an additional value.

## 2. Methods

### 2.1. Study Population and Patient Selection Criteria

The “Cardiovascular Imaging and Biomarker Analyses” (CIBER) study (clinicaltrials.gov identifier: NCT03074253) represents a clinically prospective, controlled, and monocentric study conducted at the University Medical Center Mannheim, Germany. The research adhered to the principals outlined in the Declaration of Helsinki and was approved by a regional ethics committee. Written informed consent was obtained from all patients. For the present study, patients undergoing CCTA during routine clinical care were included prospectively at the University Medical Centre Mannheim (UMM), Germany. The study was carried out according to the principles of the Declaration of Helsinki and was approved by the local ethics commission II of the Faculty of Medicine Mannheim, University Heidelberg, Germany. All patients were indicated for CCTA due to a low to intermediate PTP of 15–50% presenting with typical or atypical angina pectoris. Patients suffering from an acute myocardial infarction were excluded. Patients with severe chronic kidney disease being defined as an estimated glomerular filtration rate (eGFR) below 40 ml/min were excluded. To determine the eGFR, the Modification of Diet in Renal Disease (MDRD) formula was used.

### 2.2. Coronary Computed Tomography Angiography (CCTA)

All patients were examined by using 2 × 192 slice third-generation dual-source CT system (Force; Siemens Healthineers, Forchheim, Germany). Prior to CCTA, all patients underwent a noncontrast-enhanced cardiac CT for the evaluation of coronary calcifications using the Agatston method. The CCTA technique was chosen individually for each patient depending on heart rate and/or rhythm and body mass index, with the goal of minimizing radiation exposure while maximizing diagnostic image quality. Image acquisition techniques included traditional retrospective electrocardiographic (ECG) gating with default use of ECG-dependent tube current modulation, prospective ECG triggering, and prospectively ECG-triggered high-pitch spiral acquisitions. Tube voltage was selected by using anatomic based automated tube voltage selection (Care kV, Siemens Healthineers, Forchheim, Germany) in combination with automated tube current modulation. In the absence of contraindications, patients received 0.4 mg of sublingual nitroglycerin before image acquisition. B-blockers (5–20 mg intravenous metoprolol tartrate) were used to lower heart rates to less than 65 beats per minute in patients undergoing prospectively ECG-triggered high-pitch spiral acquisitions.

Contrast enhancement was achieved by using a bolus-tracking protocol where image acquisition began once a threshold of 100 HU had been exceeded within a region of interest in the descending thoracic aorta. A volume of 80 cc of iomeprol (Iomeron 400; Bracco Altana Pharma, Konstanz, Germany) was used with a fixed flow rate of 5 ml/s through an 18-gauge intravenous antecubital catheter, followed by 50 ml of saline at the same flow rate.

Noncontrast enhanced calcium scoring studies were reconstructed at a section thickness of 3 mm by using a dedicated algorithm (Qr36 Siemens Healthineers, Forchheim, Germany). The calcium score was calculated by using dedicated software according to the Agatston method. All contrast-enhanced CCTA data were reconstructed with a section thickness of 0.5 mm in the axial plane with a third-generation iterative reconstruction technique (ADMIRE, Siemens Healthineers, Forchheim, Germany) and a corresponding vascular algorithm (Bv40).

### 2.3. CCTA Data Analysis

All CCTA studies were evaluated on a 3D workstation (Multimodality Workplace, Syngo Via Siemens Healthineers Forchheim, Germany) using standard MPR as well as centerline curved MPR. Observers were blinded for biomarker levels. In the first step, the number of plaques that could be identified on CT images in the correct location was recorded to assess the sensitivity of different atherosclerotic plaque types. Those plaques, which were found on CT, were further analyzed. Finally, the attenuation within the coronary artery lumen was measured in three ROIs, as described previously [16].

### 2.4. CT-Based Plaque Analysis

Plaque analysis was performed offline using dedicated software (Syngo VA21, Circulation Plaque Analysis; Siemens Healthineers). Window level and width were determined using a standard window level setting. The study population was divided into four different groups—patients who did not show any sign of plaque or stenosis (no CAD), patients with noncalcified plaques defined as lesions without any detectable calcification, patients with calcified plaques defined as lesions with 50% or greater calcium, and patients with mixed plaques classified as lesions with calcification less than 50% [25].

### 2.5. Blood Sampling Procedures and Biochemical Analyses

All expressed biomarkers were measured in the serum of patients' blood. Within 24 hours before or after the CCTA, peripheral venous blood samples were taken from each patient and collected in serum monovettes® tubes and centrifuged at 2500 ×g for 10 minutes at 20°C. The aliquoted samples were cooled down with liquid nitrogen before being stored at −80°C until analysis. The whole processing took part within two hours after blood extraction. After thawing, the samples were mixed gently by inverting and centrifuged at 2500 ×g for 10 minutes at 20°C for troponin T and N-terminal probrain natriuretic peptide (NT-proBNP) analysis. Troponin T was measured with the troponin T hs STAT assay on a cobas e 602 analyzer (Roche Diagnostics, Mannheim, Germany). The limit of blank (LoB) for this assay was 0.003 ng/ml and the limit of detection (LoD) 0.005 ng/ml as described in the instructions for use [26]. For TnI measurement, the samples were gently mixed by inverting after thawing and centrifuged for 30 minutes at 3000 ×g at 4°C. Troponin I was measured with the STAT high sensitive troponin I assay on an Architect i1000 analyzer (Abbott, Wiesbaden, Germany) with a LoB of 0.7–1.3 pg/ml and a LoD of 1.1–1.9 pg/ml [27]. NT-proBNP was measured with the proBNP II STAT assay on a cobas e 602 analyzer (Roche Diagnostics, Mannheim, Germany). The LoD for this assay was 5 pg/ml [28]. Creatinine, cholesterol, low-density lipoprotein cholesterol (LDLC), high-density lipoprotein cholesterol (HDLC), triglycerides, and uric acid were measured on the cobas c 702 analyzer (Roche Diagnostics Mannheim, Germany) also after thawing, gently mixing, and centrifugation at 2500 ×g for 10 minutes at 20°C.

### 2.6. Statistical Analysis

Data were analyzed using the software IBM-SPSS version 22.0. Categorical variables are expressed as absolute numbers and percentage, whereas continuous variables are shown as mean and range. All biomarkers are presented as the median and interquartile range. For univariate correlations, we used the Spearman-Rho test. To analyze the relation between CAD groups and biomarkers, the nonparametric Kruskal-Wallis Test was used because the values did not show a normal distribution. To test the presence of a Gaussian distribution, the Kolmogorov-Smirnov test was applied. Receiver-operating characteristic curves (ROC) with the area under curves (AUC) were generated to associate hsTn and NT-proBNP with groups of patients with different plaque types. Multivariate regression models were calculated with backward elimination (Forrest plot). The odds ratios for hsTns and cardiovascular risk factors were calculated by binomial logistic regression. All analyses were considered significant when *p* was <0.05.

## 3. Results

### 3.1. Baseline Characteristics

A total of 99 patients was included prospectively with their baseline characteristics being outlined in Table 1. Mean age was 60 years, and gender was distributed evenly. About one fifth of patients suffered from diabetes mellitus (17%) or had a known cardiac family history (23%). Most of the study patients (32%) fell into the “overweight” category, with more than a third of them suffering from obesity and hypercholesterolemia. Arterial hypertension was seen in the majority of the patients (57%), and 34% were smokers. 18% suffered from atrial fibrillation, mostly paroxysmal and persistent forms.

Median left ventricular ejection fraction (LVEF) being measured by functional computed tomography was normal with 62%. 57% of the patients were found to have a coronary artery disease (CAD) that was morphologically relevant as being assessed by CCTA, whereas 43% had no signs of CAD and no evidence of CT-based coronary arterial plaques. 9% revealed noncalcified plaques, 28% showed calcified plaques, and 19% had mixed type plaque lesions with calcified and noncalcified amounts. In the present cohort, measurable concentrations of hsTnI were present in all 99 patients (100%) and of hsTnT in 78% (77/99) of the study patients.

### 3.2. hsTnI and hsTnT Differentiate Noncalcified, Calcified, and Mixed Coronary Plaques

**Figure 1:**
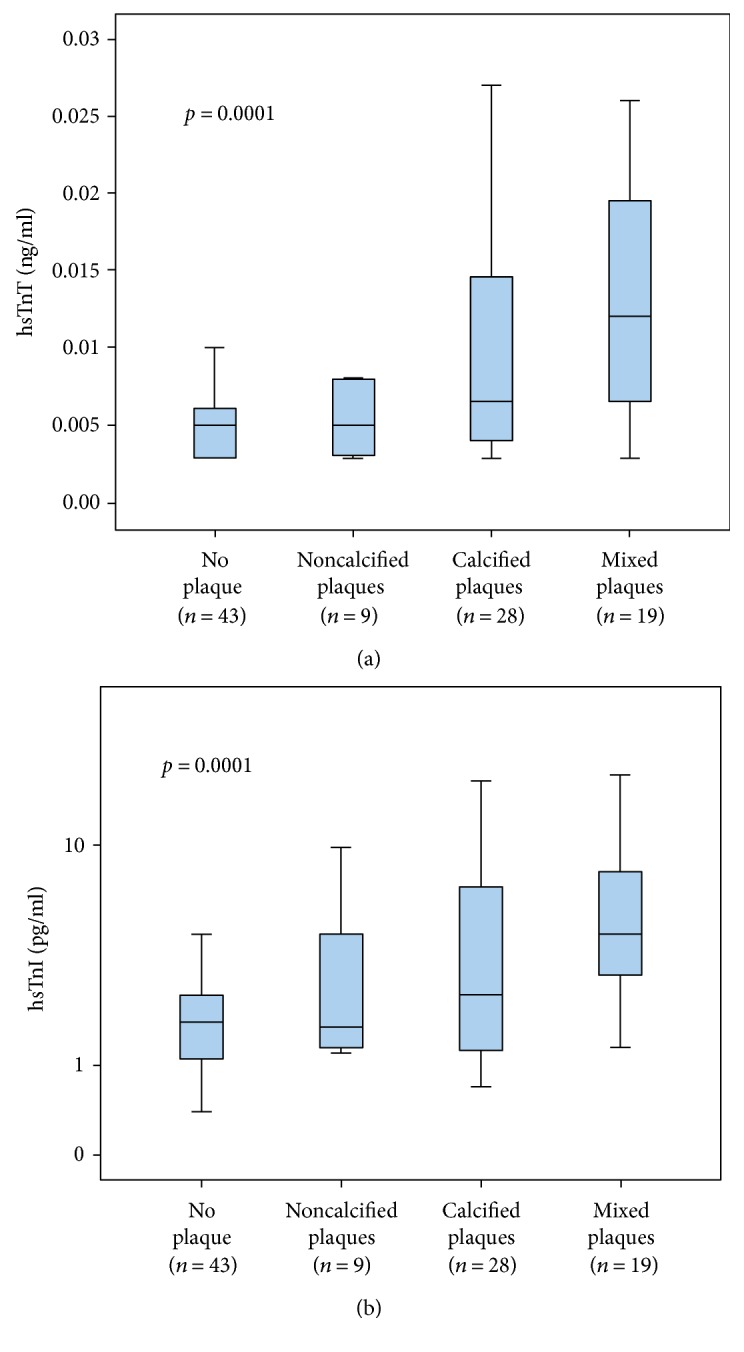
Boxplots illustrating significantly increasing levels of hsTnT (a) and hsTnI (b) in the following groups: no plaques, noncalcified plaques, calcified plaques, and mixed plaques.

As presented in Figures 1(a) and 1(b), concentrations of both hsTn were able to differentiate significantly (*p* = 0.0001) between the different groups of patients without coronary plaque and with noncalcified plaques, calcified plaques, and mixed plaques.

Slight increases of hsTnI and hsTnT were already detectable in patients without coronary plaque (*n* = 43). Highest hsTn levels were found in patients with mixed coronary plaques (*n* = 19: hsTnT, median = 0.0120 ng/ml; IQR [0.0060–0.030 ng/ml]; hsTnI, median = 4.5 pg/ml; IQR [2.9–8.5]), followed by decreasing levels in patients with calcified plaques (*n* = 28; hsTnT, median = 0.0065 ng/ml; IQR [0.0040–0.0145]; hsTnI, median = 2.5 pg/ml; IQR [1.3–7.0]), and with noncalcified plaques (*n* = 9; hsTnT, median = 0.0050 ng/ml; IQR [0.0030–0.0080]; hsTnI, median = 1.7 pg/ml; IQR [1.3–4.5]).

Cholesterol, HDLC, LDLC, and triglycerides did not differ significantly in between the groups of different coronary plaques. Detailed biomarker measurements are given in Table 2.

### 3.3. Univariable Correlations of Biomarkers and Baseline Characteristics

As presented in Table 3, both hsTnT and hsTnI correlated significantly with several patient characteristics as well as biomarkers in univariate analyses, such as age, BMI, creatinine, NT-proBNP, total cholesterol, LDLC, HDLC, and uric acid (*p* < 0.05). No significant correlations were found between hsTns and calcium and triglycerides.

### 3.4. hsTn Discriminates the Presence of Mixed Coronary Plaques

**Figure 2:**
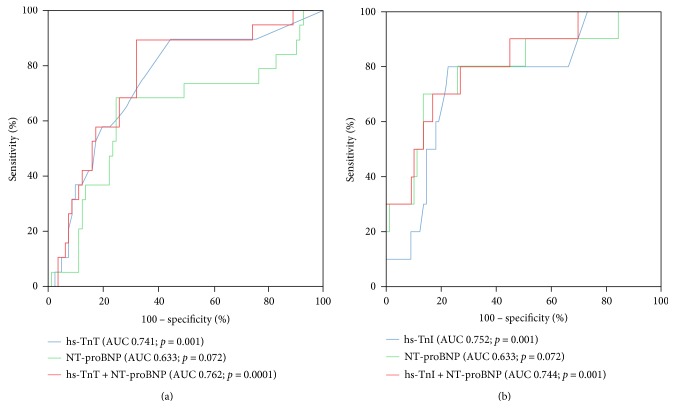
Receiver operating characteristic (ROC) curves demonstrating the capacity of hsTnT (a) and hsTnI (b) to discriminate significantly coronary artery plaques with mixed calcification.

As presented in Figures 2(a) and 2(b), both hsTnT and hsTnI were able to discriminate the presence of mixed coronary plaque composition being assessed by CCTA (hsTnI, AUC = 0.752; hsTnT AUC = 0.741; *p* = 0.001) and were significantly higher than the AUC of NT-proBNP (NT-proBNP, AUC = 0.633; *p* = 0.072). Combining hsTnT plus NT-proBNP revealed the highest AUC compared to each biomarker alone (hsTnT + NT-proBNP; AUC = 0.762; *p* = 0.0001), whereas the AUC of hsTnI plus NT-proBNP did not reveal improved discrimination over hsTnI (hsTnI + NT-proBNP, AUC = 0.744; *p* = 0.001).

### 3.5. Multivariable Regression Models Predicting Mixed Coronary Arterial Plaques

Both hsTns were adjusted within multivariable logistic regression models to predict the presence of patients with mixed coronary plaques. Besides the inclusion of hsTns, multivariable models comprised nine clinical known risk factors for CAD, such as age, gender, creatinine, LDLC, arterial hypertension, cardiac family history, smoking, diabetes, and log NT-proBNP.

Table 4 demonstrates that patients with increasing hsTnT value per each log unit were up to 9 times more likely to have mixed coronary plaques (odds ratio (OR) = 8.968; 95% CI 1.999–40.241; *p* = 0.004) even after adjusting for all of these cardiovascular risk factors. Noteworthy, log-transformed hsTnI levels were no longer associated with the presence of coronary mixed plaques in this multivariable regression model (OR = 2.390; 95% CI 0.699–8.177; *p* = 0.165) (Table 4).

## 4. Discussion

The present study evaluates whether the concentration of hsTnI and hsTnT correlates with the presence of noncalcified, calcified, and mixed coronary artery plaque composition in patients with suspected CAD being detected by CCTA. It was shown that both hsTnI and hsTnT differentiated significantly between patients with no coronary plaques, noncalcified, calcified, and mixed coronary plaques. Noteworthy, the combination of hsTnT and NT-proBNP best discriminated the presence of mixed coronary artery plaques. In multivariable logistic regression models, only hsTnT was still significantly associated with the presence of mixed coronary plaques, whereas hsTnI was not.

These findings are in accordance with the previous studies [29–31] showing valuable discrimination of hsTnT only for different CCTA-based plaque morphologies. This study demonstrates further that both hsTns T and I are able to differentiate especially the mixed plaque type, which is known to be a good indicator for the vulnerability of coronary plaques. Pundziute et al. demonstrated that the high-risk thin-cap fibroatheroma, detected by IVUS, was most frequently located in CT morphological mixed plaques [32]. In another prospective study, in patients with known and suspected CAD, it was shown that mixed plaques were an independent predictor for future cardiac events such as cardiac death, nonfatal infarction, or revascularization in a follow-up of 16 months [33]. These findings were confirmed in a multicenter prospective study in over 500 individuals with suspected CAD over a mean follow-up of 22 months [34]. Mixed coronary plaques are associated mostly with high-risk thin-cap fibroatheroma, which were shown to reveal higher circulating hsTnT concentrations in stable CAD patients, as confirmed within the present study [35]. Combining hsTnT plus NT-proBNP revealed the highest AUC compared to each biomarker alone, which indicates that this combination might yield the most promising value regarding the prediction of mixed plaques. In a large prospective study, NT-proBNP was associated with the occurrence of major vascular events and death in individuals without known CAD at baseline. Beatty et al. could demonstrate within the “Heart And Soul” cohort that a risk model containing hsTnT and NT-proBNP, combined with urine albumin-to-creatinine ratio (uACR) and current smoking, was able to better discriminate patients with stable CAD and an increased risk for a first acute myocardial infarction [36]. Taking these findings and the results of the present study into account, the combination of NT-proBNP and hsTnT might be able to discriminate patients with mixed plaques containing an increased risk for rupture.

Not only mixed calcified plaques but also noncalcified plaques are known as high-risk plaques being prone to rupture [33, 34, 37–39]. In the present study, hsTn levels were lowest in patients with noncalcified plaques, which were also shown earlier by Altintas et al. and Caselli et al. demonstrating no significant association between hsTnT and CCTA-defined noncalcified coronary plaques in patients with stable chest pain [29, 31]. However, these studies used a simplified methodological approach. They did not find an association of noncalcified coronary plaques with the “low hsTnT-group” or “high hsTnT-group” (corresponding to hsTnT values all below or above the study specific median) [29, 31]. However, the present study applies to all of the known different coronary plaque morphologies (i.e., noncalcified, calcified, and mixed plaque) and applies the numerical results of hs troponins without a too early dichotomous simplification.

Furthermore, calcification is known to occur more likely in advanced stages of atherosclerosis [17, 19, 40]. Therefore, it may be assumed that noncalcified coronary plaques might represent less advanced stages of atherosclerosis with even less coronary stenosis, revealing only slight increases of hs troponins. In contrast, Korosoglou et al. showed in a prospective study including 180 patients being suspected for CAD that an increase of hsTnT was observed in patients with noncalcified lesions [30]. However, the latter result might have been influenced by the missing differentiation between noncalcified and mixed coronary plaques, which is in contrast to the present study. Notably, the classification of Saremi and Achenbach defined noncalcified coronary plaques as containing less than 80% of calcification, which is however in contrast to commonly known classification systems allocating noncalcified coronary plaques to high-risk lesions [25]. Therefore, this present study accomplishes prior studies demonstrating a decrease of hs troponins in patients with noncalcified, high-risk plaques.

Being specifically expressed in myocardial muscle, cells troponin I and T are released from the myocardium due to cardiac injury (from ischemia or various other causes). The levels of concentrations in the blood are related proportionally to the degree of damage in the myocardium, as demonstrated in a prospective single-center study in patients presenting with an ST elevation myocardial infarction [41]. But with the upraise of hsTn assays, it is possible to detect myocardial damage not only in patients suffering from ACS, but also in patients with subclinical CAD. It has been shown in low-risk patients with stable CAD that hsTnI is associated with the risk for cardiovascular death or heart failure after a follow-up of 5 years [2]. One explanation for these findings is the clinically silent plaque ruptures causing recurrent microembolisms followed by myocardial damage and the chronic release of the troponin complex [30, 42]. Therefore, the detection of these vulnerable lesions, being responsible for this pathomechanism, represents one diagnostic target in clinical practice. CCTA is a noninvasive diagnostic method to document and analyze the different plaque characteristics especially unstable coronary plaques.

The present study delivers additional knowledge about the release of both hsTnT- and hsTnI-suspected CAD patients to differentiate, as well as CCTA, between different coronary plaque morphologies. To our knowledge, it is the first study demonstrating a correlation between hsTnI and CCTA-derived plaque morphology. Of note, hsTnI was, unlike hsTnT, no longer significantly associated with the presence of mixed plaques in multivariable regression models. This might be explained due to the fact that hsTnT was detectable in only 78% of the patients, whereas hsTnI was present in all patients. Therefore, the analysis within the multivariate regression might be influenced.

In conclusion, the findings of the present study are in line with previous studies, demonstrating that levels of hsTn can differentiate between the different characteristics of coronary plaques and might become potentially useful biomarkers in detecting vulnerable coronary plaques. These results accomplish the arguments concerning the discriminative capacity between hsTn and the presence of a subclinical CAD. hsTn is able to detect mild CAD and might therefore bear the potential to classify the morphology of coronary lesions with regard to their amount of calcium as a noninvasive method without radiation unlike CCTA.

### 4.1. Study Limitations

The present study is limited by its small sample size, thereby restricting propositions for the general population. However, the present study applies a step-wise statistical approach including all measured numerical hsTn results and avoids a too early simplification of statistics by using single and combined C statistics as well as univariate and multivariable regression models. Regarding the probability of hsTn to predict the plaque morphology, the present study shows further limitation since CCTA is not as precise as conventional angiography combined with imaging techniques such as IVUS and OCT. However, experienced observers and a high quality of CCTA images outweigh potential bias assuming that the actual plaque composition matches to the presented results. Classifying plaques only with regard to their amount of calcium is common in research with CCTA [43–45], but it cannot mirror the complexity of atherosclerotic plaques. To confirm the present findings, the study should be repeated prospectively including larger sample sizes and the evaluation of both hsTnI and hsTnT.

## Figures and Tables

**Table 1:** Baseline characteristics of study patients.

Characteristics	RV function (*n* = 99)
Age, mean (range)	60 (18–84.1)
Gender, *n* (%)	
Male	54 (54)
Female	46 (47)
Cardiovascular risk factors, *n* (%)	
Arterial hypertension	56 (57)
Hypercholesterinaemia	39 (39)
Cardiac family history	23 (23)
Smoking	34 (34)
Diabetes mellitus	17 (17)
Adipositas	32 (32)
Laboratory parameters, median (IQR^a^)	
Creatinine, *μ*mol/l	75.14 (64.5–87.5)
eGFR ml/min	88.19 (72.8–100.99)
Total cholesterol, mmol/l	4.81 (4.1–5.6)
LDL cholesterol, mmol/l	3.08 (2.5–4.1)
HDL cholesterol, mmol/l	1.24 (1.0-1.6)
Triglyceride, mmol/l	1.45 (0.9–2.0)
Uric acid *μ*mol/l	291.45 (249.8–356.9)
hsTnI, pg/ml	2.2 (1.3–4.5)
hsTnT, ng/ml	0.006 (0.003–0.01)
Atrial fibrillation	
Paroxysmal	8 (8)
Persistent	8 (8)
Permanent	2 (2)
Coronary artery disease	
1 vessel disease	16 (16)
2 vessel disease	8 (8)
3 vessel disease	2 (2)
Coronary artery disease	
None	43 (43)
Nonobstructive (<50%)	30 (30)
Obstructive (>50%)	26 (26)
COPD	7 (7)
Cancer	19 (19)
pAD	4 (4)
LVEF, median (IQR^a^)	62 (52–68)

^a^interquartile range. eGFR: estimated glomerular filtration rate; LVEF: left ventricular ejection fraction; COPD: chronic obstructive pulmonary disease; pAD: peripheral arterial disease.

**Table 2:** Median concentrations of biomarkers in all patients as well as in subgroups of CAD.

	No plaques (*n* = 43)	Noncalcified plaques (*n* = 9)	Calcified plaques (*n* = 28)	Mixed plaques (*n* = 19)	*p* value
hsTnT, ng/ml	0.005 (0.0029–0.006)	0.0050 (0.0030–0.0080)	0.0065 (0.0040–0.0145)	0.0120 (0.0060–0.030)	**0.0001**
hsTnI, pg/ml	1.8 (1.1-2.5)	1.7 (1.3-4.5)	2.5 (1.3–7.0)	4.5 (2.9–8.5)	**0.0001**
NT-proBNP, pg/ml	77.21 (43.88–140.5)	44.30 (34.93–141.10)	132.45 (45.48–1176.00)	292.10 (39.78–656.90)	**0.053**
Creatinine, *μ*mol/l	75.14 (63.7–84.0)	74.23 (55.7–89.3)	73.37 (66.3–81.3)	80.44 (72.5–95.5)	0.537
Uric acid, *μ*mol/l	279.56 (243.9–315.2)	285.50 (243.9–333.1)	306.32 (249.8–353.9)	368.78 (279.6–458.0)	**0.018**
Cholesterol, mmol/l	5.26 (4.4-5.8)	4.48 (4.1–5.9)	4.82 (4.2–5.3)	4.40 (3.5–5.6)	0.204
LDL Cholesterol, mmol/l	3.34 (2.7–4.0)	2.82 (2.6–3.2)	3.11 (2.5–3.6)	2.36 (2.0–3.9)	0.186
HDL Cholesterol, mmol/l	1.37 (1.1–1.7)	1.01 (0.9–1.3)	1.27 (1.1–1.5)	1.19 (1.0-1.5)	0.105
Triglyceride, mmol/l	1.53 (0.8–2.0)	1.58 (1.3–1.8)	1.29 (1.2–1.9)	1.68 (1.3–1.9)	0.740

hsTnT: high sensitivity troponin T; hsTNI: high-sensitivity troponin I; NT-proBNP: N-terminal probrain natriuretic peptide; IQR: interquartile range. Data are given as follows: medians (interquartile range).

**Table 3:** Univariable correlations of hsTnI and T.

	hsTNT		hsTNI	
	*r*	*p*	*r*	*p*
Age	0.458	**0.0001**	0.423	**0.0001**
BMI	0.021	0.839	−0.063	0.534
Creatinine	0.426	**0.0001**	0.434	**0.0001**
NT-proBNP	0.544	**0.0001**	0.499	**0.0001**
Cholesterol	−0.299	**0.003**	−0.288	**0.004**
LDLC	−0.288	**0.004**	−0.335	**0.001**
HDLC	−0.195	0.053	−0.201	**0.047**
Triglyceride	0.027	0.788	0.030	0.770
Uric acid	0.402	**0.0001**	0.372	**0.0001**
Calcium	−0.190	0.058	−0.090	0.371

BMI: body mass index; NT-proBNP: N-terminal probrain natriuretic peptide; LDLC: low-density lipoprotein cholesterol; HDLC: high-density lipoprotein cholesterol. Bold values indicate statistically significant *p* values (*p* < 0.05).

**Table 4:** Multivariate regression analyses for patients with mixed coronary plaques.

	hsTNT			hsTNI		
	OR	95% CI	*p* value	OR	95% CI	*p* value
Age	1.036	0.989–1.085	0.140	1.070	1.020–1.121	**0.005**
Gender	2.268	0.665–7.733	0.191	3.012	0.953–9.517	**0.060**
Creatinine	0.154	0.010–2.306	0.175	0.241	0.019–3.088	0.274
LDLC	1.000	0.984–1.016	0.978	1.001	0.985–1.017	0.906
Arterial hypertension	0.837	0.247–2.841	0.776	0.816	0.242–2.757	0.744
Cardiac family history	1.303	0.261–6.497	0.747	0.942	0.197–4.497	0.940
Smoking	0.811	0.220–2.989	0.753	0.789	0.222–2.803	0.714
Diabetes mellitus	1.070	0.208–5.501	0.936	1.335	0.272–6.559	0.722
log proBNP	0.708	0.216–2.320	0.568	0.863	0.265–2.814	0.807
log hsTNT	8.968	1.999–40.241	**0.004**	—	—	**—**
log hsTNI	—	—	—	2.390	0.699–8.177	0.165

OR: odds ratio; CI: confidence interval.
